# Parents’ experiences of paediatric end-of-life care in the UK: a multisite qualitative study

**DOI:** 10.1136/spcare-2025-005427

**Published:** 2025-07-23

**Authors:** George Peat, Emma Victoria McLorie, Laura Barrett, Helen Weatherly, Sebastian Hinde, Gabriella Lake Walker, Jane Noyes, Sam Oddie, Chakrapani Vasudevan, Richard Feltbower, Bob Phillips, Catherine Elizabeth Hewitt, Richard Hain, Gayathri Subramanian, Andrew Haynes, Lorna Fraser, Fliss Murtagh, Julia Hackett

**Affiliations:** 1 Department of Social Work, Education and Community Wellbeing, Northumbria University, Newcastle upon Tyne, UK; 2 Department of Health Sciences, University of York, York, England, UK; 3 School of Healthcare, University of Leeds, Leeds, UK; 4 Centre for Health Economics, University of York, York, UK; 5 Parent Project Advisory Group, Hebden Bridge, UK; 6 School of Medical and Health Sciences, Bangor University, Bangor, UK; 7 Neonatal Unit, Bradford Hospitals National Health Service Trust, Bradford, UK; 8 Bradford Teaching Hospitals NHS Foundation Trust, Bradford, Bradford, UK; 9 Leeds Institute for Data Analytics; Child Health Outcomes Research at Leeds (CHORAL), University of Leeds, Leeds, UK; 10 Centre for Reviews and Dissemination, University of York, York, UK; 11 Leeds Children’s Hospital Leeds Centre for Newborn Care, Leeds, England, UK; 12 Noah's Ark Children’s Hospital for Wales, Cardiff, Wales, UK; 13 PCC, Royal Manchester Children’s Hospital, Manchester, Manchester, UK; 14 The Paediatric Palliative Care Research Group, University of York, York, UK; 15 Cicely Saunders Institute, King’s College London, London, UK; 16 Wolfson Palliative Care Research Centre, University of Hull, Hull, UK

**Keywords:** Palliative Care, Paediatrics, End of life care

## Abstract

**Objectives:**

Despite the marked improvement in child mortality over the last two decades, more than 7 million infants, children and young people still die worldwide every year. In the UK, four National Health Service settings care for more than 60% of the children who die each year: neonatal and paediatric intensive care units and children and teenager cancer principal treatment centres. There is limited evidence on how end-of-life care is experienced by parents and how this differs across settings. We aimed to explore parents’ experiences of receiving end-of-life care for their child in these settings.

**Methods:**

A multisite qualitative study involving in-depth interviews with bereaved parents, analysed using reflexive thematic analysis. Recruitment via 14 National Health Service sites, three children’s hospices and two third sector organisations across the UK.

**Results:**

55 parents participated (37 mothers, 18 fathers), representing 44 children and young people (median age 7 years, range 0–23 years). 42 interviews were conducted. Experiences of care were highly variable. Parents' perceptions of high quality end-of-life care were highlighted within three themes: (1) building the foundations for high quality end-of-life care; (2) working together towards best decisions and care and (3) continuing care after death and into bereavement.

**Conclusions:**

Bereaved parents’ experiences of care at the end of life are too inconsistent. Feeling heard is crucial; without it, there is no foundation on which adequate end-of-life care can be built. Care must be tailored to the circumstances of each family and should continue after a child’s death and into bereavement.

**WHAT IS ALREADY KNOWN ON THIS TOPIC?**

- Although 4870 infants and children die in the UK each year, evidence gaps remain in how end-of-life care delivery differs across settings.

**WHAT THIS STUDY ADDS**

- Despite published guidance and pathways, delivery of care at the end of life is still highly variable across the UK.
- Parents are partners in care at the end of their child’s life. Being heard and informed is fundamental to high-quality care at the end of life; therefore, decisions must be tailored to each family’s circumstances.

**HOW THIS STUDY MIGHT AFFECT RESEARCH, PRACTICE OR POLICY**

- Frameworks, pathways and guidance on the delivery of paediatric end-of-life care need to be underpinned by higher-quality evidence, including children’s preferences and parents’ own experiences, and implemented as intended in practice.
- Care needs to continue after death and into bereavement with flexible support for families, which is able to respond to the specific needs of an individual child’s family.

## Introduction

 More than 7 million infants and children die worldwide every year,^1^ with more than 4870 dying every year in the UK.^2^,^4^ These children should receive high-quality palliative care. End-of-life care is an important component of this and refers to care and support for children and their families in the last days, weeks, months or year of life.^5 6^ While high-quality end-of-life care is important, the availability of and access to this type of care differ across countries, and there is little evidence on how this care should be delivered.^7^

While the UK has been a pioneer in paediatric palliative care, provision is inconsistent and incoherent.^8^ Although there are differences in the ways in which this care is delivered, supporting parents while their child dies should be a fundamental part of care.

In the UK, there is guidance advocating offering a choice of place of care. However, we know that most children still die in hospital, and there is scant evidence of achievement against any of the ambitions for children’s palliative care.^9^ Bereaved parents are at higher risk of poor mental and physical health outcomes than people bereaved under other circumstances.^10^ If published guidance is to bring about anticipated improvements in outcomes, it must be underpinned by high-quality up-to-date evidence based on evaluation of this type of care. Internationally, various standards of care and guidelines have been developed to improve the quality of paediatric palliative care; for example, International Meeting for Palliative Care in Children's Standards and Procedures (for paediatric palliative care in Europe),^11^ the GO-PACCS project (Global Overview-Paediatric Palliative Care Standards),^12^ National Paediatric Palliative Care Clinical Guidelines, New Zealand^13^ and the National Hospice and Palliative Care Organisation's Standards for Paediatric Palliative Care, USA.^14^ In the UK, several national organisations have published standards, frameworks, guidance and pathways for paediatric palliative and end-of-life care, including Together for Short Lives, the National Institute for Health and Care Excellence and the Royal College of Paediatrics and Child Health (RCPCH).^715^,^18^ Most, however, are based on professional opinion and draw on low quality empirical evidence.^7^

Parents are present throughout their child’s whole journey through care, from diagnosis to death and beyond, and therefore have crucial knowledge and insight about parts of the system, which professionals alone do not have. It is essential that future guidelines are underpinned by high-quality evidence, which, critically, must include the voices of bereaved parents themselves. A recent qualitative evidence synthesis showed that where healthcare teams acknowledged parents as expert partners throughout a child’s illness, offering honest, clear and tailored information, parents reported more positive experiences of care at the end of their child’s life.^19^ However, there is little evidence of how delivery of care at the end of life differs across care settings in the UK. Furthermore, what evidence there is overwhelmingly represents the perspective of mothers rather than fathers.^19^ The extent to which published care pathways for planning at the end of life and for bereavement support have been implemented in clinical practice is also unclear.^18^ The aim of this study was to examine this through exploring parents’ experiences of receiving end-of-life care for their child within United Kingdom National Health Service settings.

## Methods

This paper reports the second workstream of a wider programme of research on end-of-life care for infants, children and young people.^20^ Within this workstream, we conducted a multisite qualitative study involving in-depth interviews with bereaved parents. This builds on our earlier findings of how previously identified components of care operate in routine practice, enabling the comparison of data across different care settings.^21^ A phenomenological approach guided all aspects of this study.^22^ We used the Consolidated Criteria for Reporting Qualitative Research and reflexive thematic analysis reporting guidelines^23^ to report this study.

### Patient and public involvement (PPI)

We worked collaboratively and in partnership with a Parent Advisory Panel of 12 bereaved parents with a diverse range of experiences, throughout all research stages, from grant application through to dissemination. They were also represented on the Study Steering Committee. A bereaved parent (GLW, coauthor) helped shape the design and delivery of the study in depth, including contributing to data analysis.

### Setting and recruitment

Provision of palliative care for children and young people in the UK often relies heavily on individual health professionals and third-sector organisations,^24^ particularly children’s hospices. This means there is an inequity in provision, and in some areas, it is unclear what is being provided, by whom and how.

Children with life-limiting conditions are frequently admitted to hospital within the last year of their life, with more than 70% of children in the UK dying in hospital settings,^25^ although admissions vary significantly across the type and number of conditions.^26^ In the UK, four National Health Service settings account for the care of more than 60% of children in the last year of their life^27^,^30^ and were therefore the settings focused on in this study:^20^ principal treatment centres for cancer for children (C-PTC) or teenagers and young adults (TYA-PTC) and neonatal (NICU) and paediatric intensive care units (PICU). Workstream 1 findings highlighted the ways in which these settings operationalised components of care and therefore how delivery of care differed.^21^ Therefore, sites were selected based on the need to capture this variation in provision.

Health professionals working in recruitment sites identified and then discussed the study with eligible parents, either face-to-face or by telephone. Those interested were given a brief information sheet, consent-to-contact form and a prepaid return envelope addressed to the study team. Parents could also return these via email. Information packs containing an invitation letter and an information sheet were then sent by the study team to parents via post or email. These were followed up with a telephone call with the research team for eligible parents, enabling parents to ask questions and, if happy to do so, arrange the interview.

The Parent Advisory Panel advised recruiting via parent-facing organisations and social media platforms, addressing concerns about practitioner gatekeeping and broadening the scope of recruitment. Therefore, the study was also advertised via charity organisations’ social media channels, for example, closed Facebook groups and X (formerly known as Twitter). Parents who were interested contacted the study team either by telephone or email and were sent an information pack, as above.

### Sampling

Parents or legal guardians were eligible if they were aged 16+ years, whose child aged 0–25 years had died between 3 and 36 months prior to recruitment and had received end-of-life care from a neonatal or paediatric intensive care unit or a children’s or teenage and young adult cancer principal treatment centre in the UK. Purposive sampling was used to ensure representation of all UK nations and diverse experiences across settings, according to child characteristics, comprising diagnosis, age and illness duration. Each factor has been identified as potentially affecting access to end-of-life care.

### Data collection

In-depth interviews explored bereaved parents’ accounts of end-of-life care for their child. Parents could choose the mode of interview (face-to-face, telephone or video call). Where both parents wished to participate, individual or joint interviews were offered. Interviews were undertaken by four authors (EVM, LB and JH, all female; GP, male; all applied health researchers and previously unknown to participants).

Interviews (see online supplemental material for topic guide) were in two parts. Parents were first asked to tell their story from their child’s diagnosis through to after their death. Semistructured questions followed which identified and explored specific components of end-of-life care, if not sufficiently captured in the parents’ story.^20^ Components included advance care planning, choice over place of care and bereavement support. Informed consent (written/verbal) was obtained prior to the interview. Interviews were audio-recorded and transcribed verbatim. Transcripts were not returned to parents for checking. Interviewers debriefed individually after all interviews and bi-weekly as a group.

### Data analysis

Reflexive thematic analysis guided and informed each stage of analysis.^31^ Initially, the GP read and listened to interview audio recordings. Regular meetings with GP, JH, EVM, LB and GLW were held to enable reflective discussion regarding initial assumptions and thoughts. Data were inductively and deductively coded using NVivo^12^ software. Initial themes were developed through a reflexive and collaborative approach, drawing on postinterview reflections and discussion with JN and the wider research team. A priori assumptions of components of end-of-life care derived from workstream 1 were also discussed.^20^ Developed themes were refined in line with Parent Advisory Panel feedback.

### Reflexivity statement

Data collection and analysis members were predominantly female, all represented a variety of perspectives (health professionals, public health, policy, methodological) with various levels of prior exposure/knowledge in end-of-life care. External stakeholders provided additional perspectives and created a more balanced gendered team. To allow for transparency, interviewers completed reflective journals following each interview and met every 2 weeks to discuss and reflect on potential biases. These were used to aid group discussions and data interpretation.

## Results

### Sample

14 National Health Service sites, consisting of 11 paediatric intensive care units, 10 neonatal intensive care units, eight children’s principal treatment centres for cancer and seven teenage and young adult principal treatment centres for cancer units and three children’s hospices and two charities supported recruitment. Participants were recruited between September 2022 and July 2023.

In total, 169 study information packs were sent to parents, and 55 parents participated (participation rate 33%), representing 44 children. 42 interviews were conducted, of which 13 took place with both parents (mother, father) and the remainder were individual (fathers: n=5; mothers: n=24). Two interviews concerned end-of-life care for two children. Two parents were supported by other family members (the parent’s mother and the parent’s daughter). Mean interview length was 99 min (range: 49–221 min). Table 1 provides an overview of sample characteristics.

**Table 1:** Overview of sample characteristics

Age of child when died	Number
<12 months	23
1–4 years	4
5–10 years	3
11–18 years	9
19–25 years	5
Gender of child	
Male	25
Female	19
Gender of parent	
Male	16
Female	39
Ethnicity of parent	
White British	52
British Asian	2
Unknown	1
Any other siblings*	
Yes	30
No	14
Place of death	
Hospital setting	n=29
Neonatal intensive care unit.	15
Paediatric intensive care unit.	8
Principal treatment centre	6
Home	n=6
Referred via neonatal intensive care unit	1
Referred via paediatric intensive care unit	1
Referred via principal treatment centre	4
Hospice	n=9
Referred via neonatal intensive care unit	1
Referred via paediatric intensive care unit	4
Referred via principal treatment centre	4
Hospice referral postdeath	
Neonatal intensive care unit	1
Paediatric intensive care unit	3
Principal treatment centre	0

* Relates to whether the child had siblings at the time of the death, not at the time of data collection.

### Themes

There are three main themes: (1) *building the foundations for high-quality end-of-life care*, which has four subthemes: ensuring parents are heard, keeping parents informed, professionals being human and providing the fundamentals to enable parents to care for their children; (2) *working together towards best decisions and care*, which has three subthemes: working with parents to develop conversations about end-of-life care, offering time and space to process information and tailoring care to the individual needs of families and (3) *continuing care after death and into bereavement*, which has four subthemes: caring after death, being proactive in offering bereavement support, tailoring bereavement support to family needs and failures of the system, specific to bereavement.

## Theme 1: building the foundations for high-quality end-of-life care

### Subtheme 1: ensuring parents are heard

All parents described needing to feel listened to and acknowledged by professionals throughout their child’s care journey. Their experiences of not being heard led to a lack of trust and a need to battle with professionals to advocate for their child.

*We just felt disbelieved the wholetime, we were just arguing with them. We felt like we were battling to be listened to or believed. We didn’t trust anyone because they weren’t listening to us.* (06, NICU, mother)

Some parents also felt dismissed by professionals from the outset when they had queries about their child’s care. When this occurred, this then impacted on the foundations for relationships with professionals throughout their child’s care journey.

*The consultant burst into the room and started shouting at me and my wife that we shouldn’t be questioning the care, is essentially how it went…that set the tone that we couldn’t have that discussion anymore.* (09, PICU, father)

Some fathers also described not feeling ‘like a parent’ due to their perceived lack of inclusion in care decisions. This predominantly occurred in neonatal intensive care settings.

*If we had to make a decision, they’d listen to (mother’s name) and it was almost as if I was just there for moral support. I didn’t feel like a parent whatsoever.* (16, NICU, father)

### Subtheme 2: keeping parents informed

Parents wanted to be fully informed about their child’s care. Some spoke of positive experiences of this; for example, being regularly involved in consultant rounds or having time with professionals to ask questions and be kept up to date. Consequently, these parents felt heard and informed.

*What we valued was just being so involved in the consultants’ rounds …they would take you across and show you scans on their big monitors and explain. They would take the time to explain things.* (04, mother, NICU)

However, some spoke of being ‘kept in the dark’. This led to a heightened sense of a lack of control and a feeling that they were being expected to make decisions about their child’s care, while not being fully informed about their child’s condition.

*It’s this whole thing of you have no control in these situations; we could do nothing, there was nothing we could do actively do to help (child’s name), but not to be told what’s going on, when you know the professionals know. I always find it difficult that other people know more about my child than I’m allowed to know.* (07, mother, PICU)

### Subtheme 3: professionals being human

Parents recognised the clinical role professionals played in their child’s care. However, parents valued times when professionals displayed ‘humanness’, reflecting understanding and empathy towards their position.

*It’s the way they communicate and their mannerisms and it’s like person-centred care if that makes sense? It’s like you know them…but again we come back to the realness, being real, being a person who can identify with what’s going on*. (48, mother, TYA-PTC)

Witnessing professionals working to form bonds and connections and communicating inclusively with their child were also reflective of the ‘humanness’ valued by parents, making them feel like their child was a person who mattered.

*The palliative care team knew her so well and had such a beautiful rapport with her, they would ask her what her wishes were. She had a voice and she had choice. If I’m in hospital I need my iPad, stuff like that. It was important for us she was included in an appropriate way.* (32, mother, PICU)

### Subtheme 4: providing the fundamentals to enable parents to care for their children

Some parents benefitted from access to food, drink and accommodation and described it as enabling them to be a parent.

*The support we had at the unit was critical in enabling us to be parents. The provision of food & drink and place to stay-that really enables you to be a parent.* (23, mother, NICU)

However, experiences varied, with others recounting difficulties in accessing this provision.

*Everything’s (in hospital) focused on the child and the baby, and I get that and that is the right thing to do, but at the same time the parent neglects themselves and there’s no one looking after the parent through any of this. (Hospice) looked after the parent.* (50, mother, PICU)

Generally, specialist wards/units (eg, teenage cancer) offered the most amenable provision for both parent and child. Otherwise, support for parents in neonatal/paediatric intensive care and general wards varied substantially by location.

## Theme 2: working together towards best decisions and care

### Subtheme 1: working with parents to develop conversations about end-of-life care

Parents valued professionals working alongside them to understand the most appropriate way to discuss end-of-life care for their child. However, the role parents wished to take in decision-making varied substantially. Parents of babies cared for in neonatal intensive care were predominately first-time parents, who largely wished to be guided and led by professionals when making clinical decisions.

*It would’ve been nice for them (professionals) to be a little more decisive in what they think we should do because it put a tremendous amount of pressure on us as first-time parents in an emotional situation where your decision-making brain kind of closes off and you’re not really thinking. Afterwards, I was like ‘what if we didn’t do the right thing?* (24, mother, NICU)

By contrast, some parents whose children received care from a paediatric intensive care unit were clear on the importance of feeling in control of decisions made.

*The palliative care team were incredible around every minute decision because I wanted to feel in control and I was very honest about that.* (32, mother, PICU)

Parents of older children, predominately those with cancer, wanted professionals to work alongside them to manage how discussions about end-of-life care were conveyed to their child. A couple found navigating discussions around prognosis particularly challenging.

*I said, “(nurse specialist), he’s dying, I know that. I have done the research”. And she went, “Yes, we are going to tell him”. I went, “No, you’re not, I’m begging. Do not go in there and tell him he’s dying”. (child’s name) was like a tree hugger, he would never upset anybody, very gentle hearted*. (36, mother, TYA-PTC)

Tailoring conversations also related to the way information was relayed. Parents described how they benefitted when professionals used clear lay terminology, drew on imagery and spent time with them to ensure their understanding, with some relying on other professionals to translate complex terminology.

*And they’d (nurses) explain in layman’s terms which is massive when you’re trying to take in information that you don’t understand. And then they would come in and they would say, “That just means this.” Or, “They’re saying this,” and that’s a massive help.* (11, mother, C-PTC)

*So they got an ultrasound of what a normal brain should look like and then what (child’s name) brain looked like and sat with us and spoke through the differences…. just so that we were completely onboard with what was going on, they explained everything until we were happy.* (16, father, NICU)

Parents described how they valued professionals asking them what information they would like to be told. However, for some parents, it was clear that discussions about palliative care would not have been welcomed at earlier points of their child’s illness trajectory.

*The energy required to get through his care to the point of transplant and recovery, I don’t think it would have helped having discussions about palliative care at the same time.* (09, father, PICU)

However, others were clear they would have liked more transparency and openness about their child’s condition.

*It’s hard with parents because you don’t want to scare them. But I just wish I’d known more. I wish I’d known all the facts. I wish I’d known.* (19, mother, PICU)

It was evident that a lack of transparency impacted on parents’ ability to plan their time appropriately. This was particularly the case for fathers when trying to make decisions concerning returning to work.

*We almost got an over-positive picture and sort of one of our regrets is we could’ve spent more time with (child’s name), I went back to work to save my leave for when (child’s name) came home.* (04, father, NICU)

This lack of transparency also impacted parents’ ability to make informed decisions about their child’s treatment, with some reporting how they would have made different treatment decisions if professionals had been more open with them about their child’s quality of life or prognosis.

*If they had said, “I think (child’s name) has got 2–3 weeks to live comfortably, then I 100% would not have given him that tube because he had 2–3 weeks anyway uncomfortably. Or if they had shown us or told us what it would be like and what to expect then we would have 100% thought: we will not do that to him because it wasn’t about us.* (11, mother, C-PTC)

### Subtheme 2: offering time and space to process information

Working together with parents towards the best decisions and care also meant acknowledging and accounting for the difficult and intensive environments in which they had to process information and make decisions. Parents described how when a private room away from the unit was available, this was highly valued; however, this was not always offered.

*We were both a bit frazzled because they were doing that at her bedside. I would rather they had done that in a private room. So there are obviously other babies in the room, you can hear monitors, there’s lights flashing.* (03, mother, NICU)

Providing the necessary time, where possible, for parents to process information was recounted as important in supporting them to come to terms with substantial decisions, such as withdrawing life-sustaining treatment. When this time was not offered, parents described feeling confused and uncertain.

*And with that he (consultant) left us and we just sat there in a daze. We didn’t quite know what was going on. And then he came back and he said, “Have you made your decision yet?” And I just thought…. “I don’t know what decision they are expecting us to make, I don’t understand”. I really didn’t understand what was going on.* (11, mother, C-PTC)

Following the decision to withdraw life support, it was important to parents that they were provided time and space with their child before the process was started.

*I don’t in any way feel I needed more time, yes, I would have loved to have had more time, but I felt no pressure whatsoever in anything.* (29, mother, C-PTC)

### Subtheme 3: tailoring care to the individual needs of families

To tailor end-of-life care to the individual needs of families, professionals needed to first inform families of the options available, including where to care for their child in their final days of life. Parents of children who died in neonatal intensive care described being presented with little option of where their child died, largely because it was deemed unfeasible to move their child. Parents of children receiving paediatric intensive care were more likely to recall conversations about place of care. However, some felt the options were poorly explained, limiting their ability to make an informed choice.

*I think what could’ve been clearer at that point was them (professionals) spelling it out for us. So we had the positives and negatives of each one. Because, again, we felt a little bit lost really knowing what to do. To then be told, “Oh, you can go home”. But we weren’t told what the support was at home. And that’s why the main option was (hospice).* (50, mother, PICU)

Being at home with their child in their final days of life was the preferred option for many families, particularly where the child was older. However, a lack of community support impacted the feasibility of this option.

*Had we had the choice to have (child’s name) kept here (at home) and have people here, I would have preferred that, but there wasn’t that choice; we didn’t have nurses that could come.* (21, father, C-PTC)

Those who did care for their child at home described not always receiving the care and support they needed and the long-term impact this has had on themselves.

*I did feel very much alone making decisions that I had no clue whether I was making the right decision or not on my own. And yeah, just literally doing the very best that I could with limited knowledge and hoping that I was doing the right thing. And certainly, from my point of view, I probably wouldn’t be living with the kind of trauma that I now feel and can’t get past.* (42, mother, TYA-PTC)

Parents’ and families’ needs varied in terms of how they wished to interact and spend time with their children in their final moments of life. Some parents recounted the importance of sharing moments with their children in private, away from the intensive nature of their care setting.

*The particular doctor or nurse at the end, she really talked us through everything. We’re very thankful for that because from the moment we went into the room, you held her, like you should. Yeah, you held your baby until the last moments.* (19, father, NICU)

Others expressed a wish to remain in such environments. Parents described how tailoring care to suit these individual needs was therefore important.

*When (child’s name) was first taken off the machine (ventilator) we got asked if we wanted to go into the room and we decided to stay on the ward. I think my partner was coping better with it being more like a medical thing and he wanted nurses around him, he didn’t want it to be quiet, so were able to do that.* (27, mother, NICU)

Tailoring care also equated to working with families to ensure those who wanted to be informed were prepared about what to expect following the withdrawal of life support and the final moments of life. Those who had things explained to them in simple, clear, empathetic language appreciated this as they knew what to expect.

*But what always strikes me is the way she (clinician) explained it and she did it in layman’s terms that as a parent you recognise. She said, “Well listen, these will be the changes that you’ll see in your son, whether that’s with his breathing…”. Just through his final stages of life she was able to articulate in a way that…you know.* (40, father, TYA-PTC)

Whereas others were not so well prepared and then worried when the death of their child did not follow the trajectory they thought it would.

*We weren’t kind of prepared that there was an alternative to it happening very quickly, it just wasn’t what we were expecting. So, although it was good we had extra time with her, we were starting to panic thinking, “What if it isn’t what it seems”. So, that is one thing we wished happened at the hospital, kind of setting the expectation of, “We don’t know, it could be minutes, hours, however long.* (06, mother, NICU)

## Theme 3: continuing care after death and into bereavement

### Subtheme 1: caring after death

Following their child’s death, parents described the importance of choice regarding the circumstances in which they and their child’s body continued to receive care.

*She (child) was transported to the hospice within two hours of dying which was our wishes*. (32, mother, PICU)

Facilitating choice was reflected by parents as dependent on a degree of planning that involved parents first being informed of their options.

*So in terms of her care I know she had died but maybe the care could have been improved in terms of knowing about these types of services (after death care at a hospice). Or the fact you could take her home (after she’d died). Now I don’t know if (hospital) offers that, I was never told about that and I’m not saying I would have done it, but we just weren’t given any choices.* (10, mother, NICU)

Parents expressed how this extended into postdeath care, needing to be flexible and adaptable to individual parents’ needs. For instance, two families donated their child’s organs. In these instances, being attentive to family wishes and accommodating choices were central to parents’ experiences of the process.

*I remember being sat with (specialist nurse in organ donation) and she was saying, “Look, let’s write down, let’s have a plan of exactly what you want and how you see things happening”. We were really involved in the process and it was very much led by us. (Father) was very clear he didn’t want the monitors beeping, he didn’t want to hear him flatline and so just those little things, so the monitors were turned around and they were on mute.* (41, mother, PICU)

Some parents expressed a wish to remain close to their child following their death, while others felt less comfortable doing so.

*You know, we wasn’t allowed to really stay. We could have cuddled her and it just wasn’t allowed. They were coming in and like, “Oh, have you put her in her basket yet?”. But at the time your brain doesn’t engage, is that right? Is that how it is supposed to be? “You can go now, it’s all done”. That is exactly what she said, and we just went. You know, like a pair of divs, we just went.* (02, mother, NICU)

*Even though I didn’t want to see him, I just wanted him nearby so I knew he was safe, if that makes sense.* (16, mother, NICU)

Adapting care to different parent preferences was therefore important but not always enacted. This had a significant impact on the parents, leaving them with questions, regrets and negative experiences they carried with them forever.

*It was horrible, basically, they had to kick me out, “We’re sorry but you’re going to have to go shortly because we can’t have (child’s name) left in the room. You know, “We’ve got to wash her and get her ready” and stuff. Well why couldn’t I have washed her?* (45, father, TYA-PTC)

*The nurse who I didn’t really get on with, she said, “I’ll put her some clothes on for you,” and I said, “No, I don’t want you putting clothes on her”. And she said, “Well, she’s not going to know”, and I said, “But I’ll know” …I have got a memory card with pictures and she has got clothes on…it destroyed me because I asked them specifically not to (dress her).* (02, mother, NICU)

Care setting influenced the nature of extended time parents spent with their child. In most clinical settings, extended time, when offered, meant staying in the same room with their child. While parents who received after-death care from a children’s hospice described the option of staying in separate accommodation but near their child.

*But I got five precious days with her (at the hospice) where I could go and see her everyday. I could hold her. We could talk to her. It wasn’t in a hospital so there wasn’t any noises or beeps, you know?* (10, mother, NICU)

The nature of how parents spent time with their child’s body also varied. Some parents sourced comfort from being directly involved in the washing and dressing of their child’s body. Other parents preferred such activities to be undertaken by the care team. In either case, it was vital that parents’ wishes were heard and acted on by the care team.

*So they gave him a bath and they said, “Look, we’ll give him a nice bed bath and get him nice and clean and everything”. So I sat in the other room and it was so lovely hearing them talking to him whilst they were washing him and it was really nice to hear that.* (52, mother, TYA-PTC)

Certain circumstances, such as a postmortem, meant parents were not able to be with their child. Consistent communication with parents during this time was described as comforting. Without this communication, parents could be left in the unknown.

*There was a post-mortem nurse who communicated with us. She’d called me twice and the second time she called me after she’d bathed him and she told me all the products she had used and she told me she had handed him over to our funeral director. So, I knew he was in really, really safe hands because just speaking to her gave me comfort.* (17, mother, PICU)

### Subtheme 2: being proactive in offering bereavement support

Parents, predominately leaving neonatal or paediatric intensive care following their child’s death, relayed being provided information on bereavement support in the form of leaflets. Few parents had the inclination or emotional energy to seek support in the initial weeks and months following their child’s death.

*‘So they give you an information pack. You get loads of booklets. If you don’t have a partner who ‘reached out to these people you have nothing. What I mean is no one makes an appointment for you, no one does anything for you. And I understand you can’t stay there forever. But it just very, you’ve given me leaflets like I’ve lost a dog, I’ve lost my child.* (10, mother, NICU)

Active referrals to bereavement support were therefore important, as was the proactive offer of support and guidance to undertake tasks such as the registration of death and arrangement of the funeral. Such support was rarely evident from most services except by bereavement charities and children’s hospices.

*So from that charity we had an immediate support officer, I had a phone call from her the second day we were out of hospital, checking if there was anything that she could do, did we need help organising the funeral, can she come and see us, gave us her number.* (27, mother, NICU)

### Subtheme 3: tailoring bereavement support to family needs

Access to bereavement support for families appeared inconsistent, including therapeutic approaches and group support, with parents describing differences and limitations in approach, duration and delivery.

*There was the option for to join I think a Facebook group, I don’t have Facebook, and there was the opportunity to do Zoom group counselling and I just didn’t feel I wanted to do group counselling. I guess that was the only option for any support, which just wasn’t suitable for us.* (23, mother, NICU)

Parents emphasised the importance of offering bereavement support to all family members.

*There’s very little sibling support out there. She would love to attend a (bereavement) group but there’s nothing for siblings her age. She can attend a normal group but then those are people like my dad’s age. There’s nothing for the early, over 18’s, kind of young adults.* (49, mother, TYA-PTC)

For those families who were engaged with a children’s hospice, they were able to access family-centred bereavement care with support available for all family members.

*(Hospice) helped brilliantly in looking after me and (father). They’ve (also) spoken to my mum. (sibling’s name) has been involved in stuff with (hospice), we’ve gone and had presents with Santa for him as well. Again, they’ve been fantastic.* (10, mother, NICU)

Greater flexibility in referral length was also outlined by parents receiving hospice bereavement care in comparison to other organisations.

*I had about ten sessions (of therapy), but they started literally just as we lost him. I tried to make the sessions last longer by not having them every week, but every month because I felt I would need them later down the line. So, I did that but obviously they ended and then there was nothing.* (26, mother, NICU)

### Subtheme 4: failures of the system, specific to bereavement

Poor coordination between services following the child’s death could result in difficult and challenging experiences for parents. These experiences ranged from mothers having to source medication for stopping lactation at a time when they were grieving to parents receiving unwelcome and distressing phone calls from services uninformed of their child’s death.

*I got a message from the health visitor, congratulations on the birth of your baby, we’re going to come out and see you. I can’t tell you how painful it is to get something like that. It’s like no one cares and no one cares you’ve had a baby and that baby has died because it doesn’t mean anything to them.* (10, mother, NICU)

The sudden withdrawal of financial support was also highlighted by some parents. Without this support, these parents had to engage in environments they did not feel ready or adequately supported to do so.

*Two days after (child’s name) died I got a letter to say that my Child Disability Living Allowance stopped, like how can they process it that quick but it took 18 weeks to set up? I think it’s just disgusting, they don’t even give you a little bit of time to try and get yourself back into the normal world*. (11, mother, C-PTC)

## Discussion

### Main findings

This is the largest and rigorously conducted UK-wide qualitative study to date evaluating parents’ experiences of end-of-life care for their child, conducted during a challenging time for the National Health Service. New theoretical insights provided by the study address existing gaps in how parents conceptualise and perceive high-quality end-of-life care for their child across settings. Despite the existence of published guidelines and frameworks for optimal paediatric end-of-life care, this study found parent experiences of care are highly inconsistent, with variations in resource and practice both within and across unit types in the UK National Health Service settings.

Our findings indicate parents perceive high-quality end-of-life care as reflective of several interconnected elements. From the outset, there is a need for open and consistent dialogue with professionals, in which parents are kept informed and their views actively sought. Second, parents wish to be acknowledged as equal partners in their child’s care, ensuring decisions about care and subsequent care delivery take into account the unique circumstances and preferences of the family. Finally, end-of-life care should continue after death and into bereavement, offering families care and support through proactive and coordinated service delivery.

This study provides crucial and novel insights into contemporary parental experiences of the delivery of paediatric end-of-life care in the UK and the extent to which published guidelines and pathways^7 15 17 18^ have been implemented across various settings. Findings reveal differences in care delivery both within and between unit types and delineate the elements that parents consider most pertinent to the delivery of high-quality end-of-life care.

Key frameworks and pathways highlight the importance of good communication with families.^7 18^ Our findings demonstrate the impact on parents when sufficient time is not given for them to make decisions and when professionals experience discomfort introducing aspects of palliative and end-of-life care concepts, including end-of-life care planning.^20^ They also echo those of previous studies, emphasising the value of honest, clear and timely information to parents.^1932^,^36^ However, our findings advance understandings around communication style. Parents’ preferences are far more nuanced and individual than previously acknowledged, with notable differences within and across unit types. For example, discussions around prognosis and end of life, particularly in relation to older children, need to be handled carefully, with professionals working collaboratively and in partnership with parents to manage these. Conversely, within neonatal and paediatric intensive care settings, parents often wished professionals had been more transparent and clearer with them about their child’s prognosis from the outset. This reinforces the importance of training and education for professionals to support them to work alongside families to enable understanding around a family’s unique position, communication preferences and supporting them in exploring uncomfortable truths.

Offering choice over the location of care at the end of life is an important example of tailoring provision to the needs of individual families.^19^ Despite best practices, tensions often arise between paediatric palliative care professionals and parents when making end-of-life decisions, with families bearing the emotional and psychological impact of these strained dynamics. Professionals do understand the importance of high-quality end-of-life care, such as providing choices relating to the location of care.^37^ However, our study shows that the provision of choice is inconsistent across geographic locations. While many parents prefer to care for their child at home, in many areas, a lack of palliative care services makes the option impossible, despite professionals being aware of the importance of providing choices at the end of life.^37^ Parents reported traumatic experiences where care in the home setting was offered without adequate support. The option of transfer to hospice care prior to, or shortly after, death depended on local patterns of referral and of practice with respect to timely discussions of the option with parents. Referrals to hospice (both pre/post death) occurred the least among patients in neonatal intensive care units. However, this was not necessarily due to preferences not being met. Choice of accommodation for parents on site was also inconsistent, with some centres offering no choice at all and others providing the option of private suites. Parents themselves need looking after, ensuring they are supported to make the best decisions for their child during their care and for themselves after their death.

Specific care standards for bereavement state that support should continue ‘throughout death and beyond’, providing all members of the family with time, privacy and ongoing access to bereavement and support in the manner and location of their preference.^7^ Our study found limited evidence that such standards are met in practice. There are inconsistencies across settings in access to and delivery of bereavement support, particularly in the longer term. Professionals have also highlighted system-level constraints impacting their ability to appropriately support families during this time and the emotional impact of their inability to provide high-quality care.^37^ RCPCH guidance on bereavement support states ‘families should be given access to (bereavement) information sources’ and ‘other forms of therapy can be offered to families requesting such help’. Our study suggests that bereavement support needed to be proactively offered since families are rarely able to source such support themselves. Parental narratives, especially those of children who died in neonatal and paediatric intensive care units, suggest a perception that units were often reluctant to make proactive referrals to providers of bereavement support. This highlights how parents are becoming aware of professionals' inability to appropriately support families during this time due to constraints within the current UK health system.^37^ Where it was offered, bereavement support tended to be limited to a specific approach and timeframe (eg, a certain number of sessions), with limited scope for repeating/continuing the support. Such a restriction is at odds with current understanding of parental grief as persistent, evolving and unique to each individual.^38^ Bereavement support provided by children’s hospices offers families a wider choice of approach and duration, but access relies on families being engaged with a children’s hospice. Our findings, therefore, complement calls for the need for robust evaluations of bereavement services.^39^

### Strengths and limitations

The study recruited a diverse sample, representative of experiences both across settings and prognosis (eg, neonates to older children who died of cancer). The study recruited fathers (n=16), addressing an under-representation of their perspective in the existing literature. However, the sample was largely White British and therefore does not consistently represent the experiences of parents from other ethnic backgrounds. Future research is therefore required to highlight and understand specific ethnic and cultural considerations and practices regarding end-of-life care. Future research also needs to consider making study materials more accessible and explore the use of translators to aid data collection. However, appropriate funding and resources are required.

### Implications for policy and practice

In the absence of high-quality empirical evidence, published frameworks, guidance and pathways for paediatric palliative and end-of-life care^715^,^18^ are largely based on expert opinion and best practice assumptions. Our study suggests that in practice, such published guidance is not being implemented in many places. Consequently, the delivery of end-of-life care in the UK is inconsistent and inadequate. The lack of choice over location of death and access to bereavement services provided particularly clear examples.

To address the evident variation in experiences of paediatric end-of-life care both within and across National Health Service settings, it is vital that frameworks, pathways and guidance are updated in line with high-quality evidence on care at the end of life, informed directly by parents’ experiences and subsequently implemented consistently in practice. Doing so will support the optimisation and standardisation of practice.

### Conclusion

There is a long way to go to further optimise and individualise end-of-life care for children, young people and their families. Policy and guideline intentions are laudable but fall down in implementation and frequently do not meet the expectations of many parents, families or professionals.^37^ Commissioners and those delivering services need to take a more proactive and family-focused approach to monitoring and evaluation to ensure that the care and services provided are child- and family-focused, delivered using a partnership model and achieve the anticipated outcomes in a value-based healthcare context.

## Supplementary material

10.1136/spcare-2025-005427 online supplemental file 1

## Data Availability

No data are available.
