# Supplementary material for: Parents’ experiences of paediatric end-of-life care in the UK: a multisite qualitative study
Source: BMJ Support Palliat Care. 2025 Jul 23;15(5):e005427. doi: 10.1136/spcare-2025-005427 (PMC12421113; doi:10.1136/spcare-2025-005427)
Supplement: online supplemental file 1 [file spcare-15-5-s001.docx]

**End of Life Care for Infants, Children and Young People** **(ENHANCE)**

Workstream 2: to investigate the experiences and outcomes for children, parents and health professionals of different models of providing end of life care

**Outline Topic Guide –Parents - Interviews**

**Overview of structure and content**

**Introducing the interview and consenting process**

- Introduce self and thank for time.
- Check feeling OK and fine to do the interview.
- Explain expect interview to last ~1.5 hours.
- Brief reminder of the purpose of the study
- Explain / remind: fine to take breaks, ask to avoid certain topics, or stop the interview, and confidentiality.
- Briefly explain overview structure of the interview.
- Check any questions and ok to proceed.
- Start recording.

**Finding out about the child and family**

Ask parent to tell you a little bit about their child and their family.

**History of the child’s condition**

Exemplar opening script: “And now, please could you tell me a bit about [name of child’s condition?...”

**The parent’s story of their experiences of palliative and end of life care for their child**

Telling the story: possible skeleton script: *“Would it be OK now if I ask you to tell me your story of what happened…?”*

**Semi-structured follow-up questions on target topic areas, if not covered in parent’s story:**

**Care in the last days, weeks and months of life**

Can you tell us about the care your child received in the last days, weeks and months of their life?

Can you tell us about any related care your child or family received before then?

Was this alongside / in conjunction with any treatment your child was receiving?

What sort of involvement did you have in what happened and the decisions / discussions about what care was provided?

How was [child] involved in these decisions / discussions? What was important to your child?

Did your child have an advance care plan?

Were you and your child offered a choice over place of care?

Have you and your family been offered bereavement support?

Can you tell us about your working arrangements during this time?

Can you tell us about any costs or expenses you might have had during this time?

Can you tell me a little bit about the hospital facilities offered/available to you during this time?

**Learning from what works less well**

Can you tell us about any experiences that could have been better?

How do you think these experiences affected your child and family?

Did this adversely impact child and family wellbeing, and if so, how/why?

What would have made these experiences more valued for [child’s name] and your family?

Are there things that the individuals who provided care in those days, weeks and months before [child’s name] died could do better?

Can you tell us about any unmet care needs your child and family had during the days, weeks and months before they died?

**Learning from what works well**

How do you think your child benefited from the care s/he received in the days, weeks and months before they died? And what about the benefits for you and other family members?

What aspects of care or care planning made a positive difference to the days, weeks and months leading up to your child’s death? What else helped during this time?

Are there any other experiences of the care you received that you particularly valued and why?

Did this positively impact child and family wellbeing, and if so, how/why?

What do you think made these experiences possible? Can you remember in detail what specific factors made them that way?

If those experiences were to become the norm, how would things need to change?

What are the things that you most valued from the individuals who provided care to [child]?

**Suggested improvements and final thoughts**

Do you have any other thoughts about how care in those last days, weeks and months can be improved for children and young people?

If there was only one thing you could change about how care in those last days, weeks and months is introduced and provided, what would it be?

Is there anything else you would like to share?

**Close:**

Moving towards end of the interview

Remind re confidentiality.

Next steps for the study

Thank you
